# *Equalizer* reduces SNP bias in Affymetrix microarrays

**DOI:** 10.1186/s12859-015-0669-y

**Published:** 2015-07-30

**Authors:** David Quigley

**Affiliations:** Helen Diller Family Comprehensive Cancer Center, University of California at San Francisco, San Francisco, CA 94158 USA

**Keywords:** Microarray, Affymetrix, eQTL, Single nucleotide polymorphism

## Abstract

**Background:**

Gene expression microarrays measure the levels of messenger ribonucleic acid (mRNA) in a sample using probe sequences that hybridize with transcribed regions. These probe sequences are designed using a reference genome for the relevant species. However, most model organisms and all humans have genomes that deviate from their reference. These variations, which include single nucleotide polymorphisms, insertions of additional nucleotides, and nucleotide deletions, can affect the microarray’s performance. Genetic experiments comparing individuals bearing different population-associated single nucleotide polymorphisms that intersect microarray probes are therefore subject to systemic bias, as the reduction in binding efficiency due to a technical artifact is confounded with genetic differences between parental strains. This problem has been recognized for some time, and earlier methods of compensation have attempted to identify probes affected by genome variants using statistical models. These methods may require replicate microarray measurement of gene expression in the relevant tissue in inbred parental samples, which are not always available in model organisms and are never available in humans.

**Results:**

By using sequence information for the genomes of organisms under investigation, potentially problematic probes can now be identified *a priori*. However, there is no published software tool that makes it easy to eliminate these probes from an annotation. I present *equalizer*, a software package that uses genome variant data to modify annotation files for the commonly used Affymetrix IVT and Gene/Exon platforms. These files can be used by any microarray normalization method for subsequent analysis. I demonstrate how use of *equalizer* on experiments mapping germline influence on gene expression in a genetic cross between two divergent mouse species and in human samples significantly reduces probe hybridization-induced bias, reducing false positive and false negative findings.

**Conclusions:**

The *equalizer* package reduces probe hybridization bias from experiments performed on the Affymetrix microarray platform, allowing accurate assessment of germline influence on gene expression.

## Background

Naturally occurring germline DNA variants in human populations and distinct strains of model organisms affect many phenotypes, including basal levels of gene expression [1–4]. When the genotype of a variant is significantly associated with expression of a nearby gene, that variant is said to tag a *cis*-acting expression Quantitative Trait Locus (*cis*-eQTL). Several groups have noted that Single Nucleotide Polymorphisms (SNPs) can interfere with hybridization of cDNA to the microarray probes that span the SNP [5–8]. This technical artifact produces spurious eQTL signals if only one strain or population subgroup bears the SNP, as the artificially lower gene expression appears to indicate a *cis*-acting eQTL.

A recent publication highlighted this serious problem which has caused incorrect findings to be reported and replicated widely in the genetics literature [8]. Ramasamy *et al.* suggested a protocol that removed microarray probes bearing SNPs, but did not provide software tools to automate this approach. Previously published packages predict the presence of SNPs that affect microarray hybridization using statistical models [7, 9, 10]. The *equalizer* package provides a general solution to this problem for the commonly used Affymetrix gene expression microarrays. *equalizer* can be applied to any experiment performed on the Affymetrix Gene or IVT platforms where founder sequences are known, including humans. In this manuscript I compare *equalizer*’s results to the performance of a recently published method for identifying probe bias by statistical measurements [10]. I demonstrate the application of *equalizer* to microarray data generated for an eQTL study of mouse skin and mammary gene expression, as well as human lymphoblastoid cell lines.

The *equalizer* package makes several contributions. First, as it uses known genomic variants to identify probes affected by SNPs, it does not require expression measurements in replicate parental samples. Such replicates are not available in human populations and may not be available in individual studies of model organisms. Second, it can remove any probe that intersects a feature specified in a Variant Call Format (VCF) file, and is not restricted to genes expressed in a particular tissue used to identify potentially affected probes. It is straightforward to prepare custom microarray annotations for any genome where variant information is available. Third, while *equalizer* is fully compatible with Bioconductor analysis tools, *equalizer* generates a new copy of the Affymetrix array definition files and can be used by any analysis pipeline.

## Implementation

**Fig. 1:**
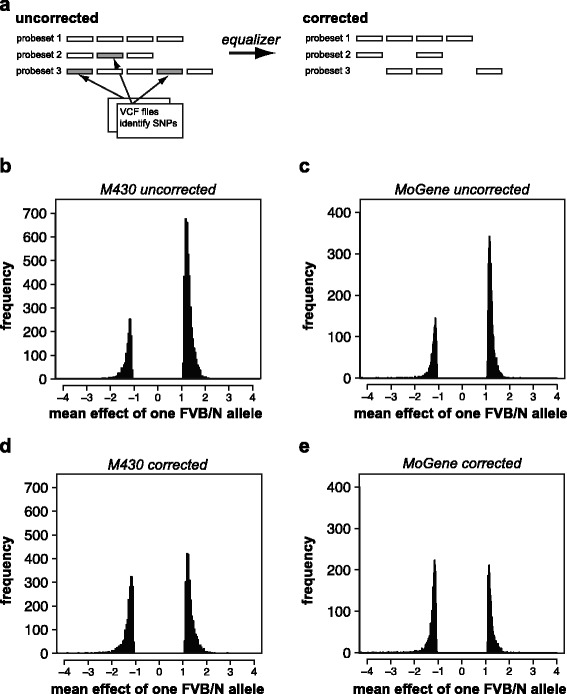
*Equalizer* reduces SNP eQTL bias. **a** Schematic illustration of *equalizer* operation. **b**,**c**,**d**,**e** Histograms of mean effect of a single mouse FVB/N allele, calculated from the difference of mean expression levels for a given gene when divided by genotype at eQTL locus. At any given eQTL all mice will be either homozygous (FVB/N, FVB/N) or heterozygous (FVB/N, SPRET/Ei). A value greater than zero indicates mice homozygous for FVB/N alleles at that locus have higher expression levels. Histograms plotted for **b** M430 uncorrected skin, **c** Gene ST uncorrected mammary, **d** M430 corrected skin, and **e** Gene ST corrected mammary

Affymetrix microarrays report a probeset expression value summarized from an ensemble of probes. The location and probeset assignments are controlled during the normalization procedure by files available from the manufacturer. *equalizer* improves the signal quality in the presence of SNPs by selectively removing probes that intersect the SNPs from their probeset assignments (Fig. 1a). To create a modified Affymetrix description file set, the user provides *equalizer* with the appropriate platform description files freely available from the Affymetrix web site (*http://www.affymetrix.com*), including a BED-formatted file describing all probe locations. The user also provides one or more VCF files specifying the location of SNPs or other genomic features that may interfere with probe hybridization. *equalizer* then uses the *bedtools* package [11] to identify probes which overlap with the location of a SNP and a software script written in Python to create a new set of internally consistent platform description files consisting of probesets where these probes have been removed. *equalizer* also produces a report indicating how many probes were removed from each probeset. If every probe in a probeset is removed by *equalizer*, the probeset itself is also removed from the new annotation files. The resulting files are suitable for use by any downstream normalization pipeline, including R’s *oligo* package [12] or the Partek Genomics Suite. *equalizer* can be invoked either from an R package (also called *equalizer*) or by calling a Python script from the command line. If called from the command line, *equalizer* produces an R script that can be used to create an R Platform Design Information package.

## Results and discussion

### eQTL analysis with *equalizer*

Genetic crosses between mouse strains with differing susceptibility to cancer are a useful tool for identifying genes which play a role in cancer biology. The goal of these studies is to map loci associated with gene expression and other phenotypes. The SNP bias problem is particularly obvious in genetic studies of model organisms where the founder strains are not evolutionarily equidistant from the reference strain against which the microarray probes were designed. The inbred mouse strain *Mus spretus* is separated by approximately two million years of evolution from *Mus musculus* strains such as C57BL/6, the reference mouse genome strain*.* We have previously performed eQTL experiments using backcrosses of the *Mus spretus* strain (SPRET/Ei) and a *Mus musculus* strain (FVB/N) [4, 13]. Backcrossed mice were generated by breeding inbred *Mus spretus* SPRET/Ei animals with inbred *Mus musculus* FVB/N animals to make an F1 generation, and then breeding F1 progeny with the FVB/N line. Genotypes at any given locus on backcross chromosomes one through nineteen are therefore expected to be 50 % heterozygous (SPRET/Ei, FVB/N) and 50 % homozygous (FVB/N, FVB/N).

Analysis of published genome sequences for the strains FVB/N and SPRET/Ei [14, 15] by *equalizer* indicated that of the 833,910 probes on the Affymetrix Gene 1.1 ST array, 176,191 intersected a SPRET/Ei SNP while only 24,196 intersected a FVB/N SNP. The imbalance between SPRET/Ei and FVB/N SNPs was more extreme on the older mouse M430 2.0 chip; of its 496,469 probes, 104,772 intersected a SPRET/Ei SNP while 10,296 intersected a FVB/N SNP. Probe-intersecting SNPs could therefore affect eQTL results for either parental genome, but were *a priori* more likely appear on the SPRET/Ei genome at a ratio of more than 7:1 for Gene ST array or 10:1 for the M430 array. An unusually large number of *cis*-eQTL with lower expression associated with the SPRET/Ei allele would strongly suggest technical bias due to probe hybridization artifacts.

To test *equalizer* I obtained 71 Affymetrix M430 2.0 microarrays measuring gene expression in mouse skin and 115 Affymetrix Gene ST 1.1 microarrays measuring gene expression of normal mouse mammary tissue [4, 16]. eQTL analysis of a backcross design tests all loci to determine whether animals heterozygous at a given locus show significantly different expression levels for a given gene compared to animals with a homozygous genotype at that locus. There are many phenotypic differences between *Mus spretus* and *Mus musculus* strains of mice [17], but there is no reason to expect *a priori* that these macroscopic differences would be consistently associated either higher or lower expression of any particular gene when that gene is derived from a SPRET/Ei allele. We therefore expect higher expression to be associated with the heterozygous eQTL allele 50 % of the time.

I identified eQTL in the skin and mammary datasets using a custom-written software package (*eqtl*) that `performs linear regression for eQTL experiments (see Methods). For each of the 16,588 probesets the *eqtl* program tested the association between probeset expression and genotype variation at each locus, reporting the statistically strongest locus as a candidate eQTL. In the skin dataset, mice bearing a SPRET/Ei allele at these loci had lower average expression of the eQTL probeset 76 % of the time (*P* < 0.001, binomial test, Fig. 1b). In the Gene ST dataset, 70 % of *cis*-eQTL had lower average expression for the SPRET/Ei allele (*P* < 0.001, binomial test, Fig. 1c). As noted above, the expected value in both cases in the absence of SNP bias was 50 %. After correction using *equalizer*, this analysis reported lower average expression for the SPRET/Ei in only 57 % of genes in the M430 experiment and 53 % of genes in the Gene ST experiment (Fig. 1c and d).

**Fig. 2:**
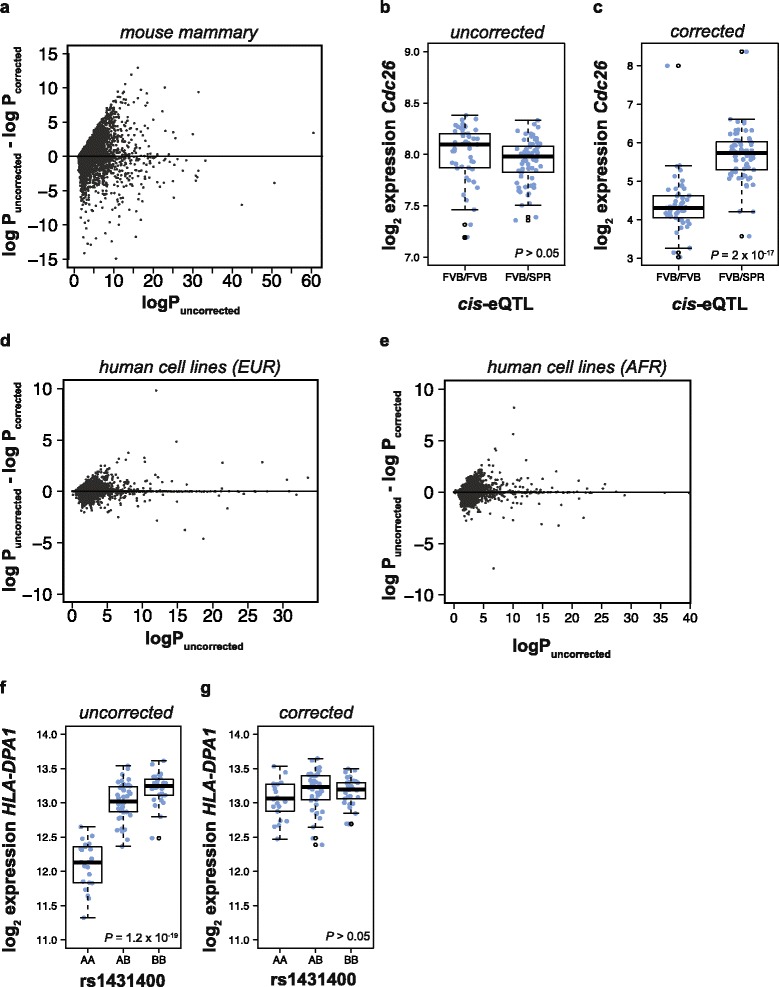
The effect of *equalizer* on eQTL results. **a** Plot of the change in eQTL statistical strength after correction *vs.* the uncorrected eQTL statistical strength for mouse mammary data. **b, c** Expression of *Cdc26* at the *cis*-eQTL locus (**b**) before correction and (**c**) after correction, showing an increase in significance after correction. Expression levels are divided between homozygous (labeled FVB/FVB) and heterozygous (labeled FVB/SPR) genotypes, where FVB indicates FVB/N and SPR indicates SPRET/Ei. *P* values indicate linear regression, box and whiskers plots indicate median and first/third quartiles. **d,e** Plot of the change in eQTL statistical strength after correction *vs.* the uncorrected eQTL statistical strength for, (**d**) human EUR data and (**e**) human AFR data. **f,g** Expression of *HLA-DPA1* in human AFR samples (**f**) before correction and (**g**) after correction, showing a decrease in statistical significance after correction. Expression labels are divided between the AA, AB, and BB versions of the rs1431400 genome variant. Plotted as 2b

After *equalizer* correction many eQTL significance values changed. In the mouse mammary data, after correction 8104 of the 15,867 probesets (51 %) decreased in statistical significance. However, considering only those probesets where the uncorrected eQTL result was significant at a FDR ≤ 0.05, 68 % of results decreased in significance after correction. Genes with higher uncorrected statistical significance were significantly more likely to have reduced significance after correction, indicating that many of the strongest results were inflated by probe bias (Pearson r = 0.20, *P* < 2 x 10^−16^). However, a large number of loci also increased in eQTL significance after correction (Fig. 2a). This indicated that in many cases probe interference artifacts were reducing the statistical significance of eQTL effects. A typical example was the *cis*-eQTL for the gene cell division cycle 26 (*Cdc26*), which increased in statistical significance after correction from *P* = 0.17 to *P* = 2 x 10^−17^ (Fig. 2b and c). Nine of the original 12 probes for this probeset were removed by *equalizer*. The remaining three probes reported lower average expression levels than the original 12, but these remaining probes should be free of SNP bias and therefore a more effective tool for detecting eQTL.

Many individual strains of model organisms have been sequenced, making it possible to have complete knowledge of the possible genotypes of their progeny. However, many human eQTL studies are conducted without exome or whole genome sequences of the participants. To test the applicability of *equalizer* to human samples where individual genome sequences may not be available, I identified SNPs with a minor allele frequency greater than 5 % in Caucasian-derived (EUR) and African-derived (AFR) genome sequences obtained by the 1000 Genomes Project [18]. This identified 6,972,489 EUR SNPs and 9,957,360 AFR SNPs. I then obtained microarray and genotype data measured in lymphoblastoid cell lines derived from 96 donors of European ancestry (EUR) and 95 donors of African ancestry (AFR) [19]. Of the 604,259 probes on the HG-U133 Plus 2.0 array, 16,849 overlapped a EUR SNP, while 26,454 probes overlapped an AFR SNP. These numbers were dramatically lower than those in the mouse genome experiments, reflecting the relative proximity of human genomes to the human reference compared to that of the mice to the mouse reference. As expected from the overall larger number of SNPs in African-derived populations compared to Caucasian-derived populations, African-derived samples had a larger number of probe-intersecting SNPs than Caucasian-derived samples. I then separately corrected gene expression for Caucasian-associated and African-associated SNPs.

In both AFR and EUR samples the observed eQTL effect sizes and the changes in eQTL significance after correction were smaller than those observed in the mouse samples (Fig. 2d and e). In a mouse genetic backcross there are only two possible genotypes, and the expected minor allele frequency is 50 %. In contrast, the minor allele frequencies in human populations varied from 5 to 50 %. However, removing probes which intersected SNPs still affected many *cis*-eQTL, and spurious SNP-associated effects could be detected. *HLA-DPA1* is a member of the major histocompatibility complex, a highly polymorphic family of genes crucial for immune cell recognition and antigen recognition [20]. Uncorrected gene expression profiles suggested a strong eQTL influencing expression of *HLA-DPA1* in African-derived samples (Fig. 2f), but this relationship was not present after removing the seven probes that intersected a SNP (Fig. 2g).

### Comparison to other approaches

**Fig. 3:**
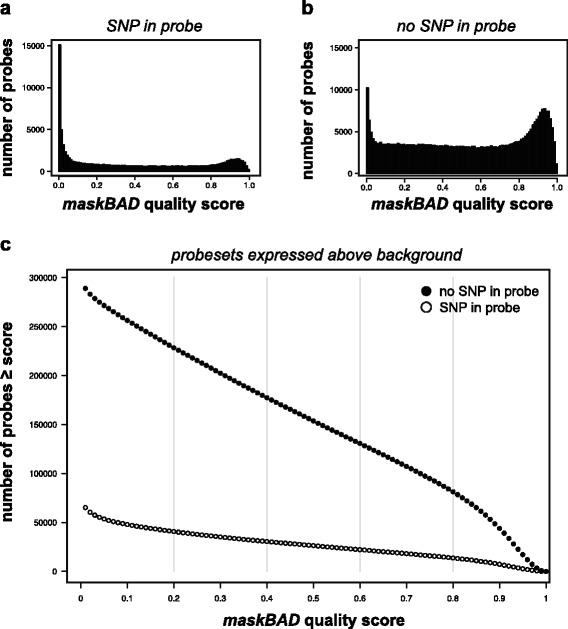
*MaskBAD* scores for probes kept or excluded by *equalizer*. **a,b** Histograms of *maskBAD* scores for probes that (**a**) intersect a SNP and (**b**) do not intersect a SNP in either FVB/N or SPRET/Ei genomes. **c** Number of probes in probesets expressed above background levels selected at *maskBAD* quality scores ranging from 0 to 1, plotted separately for probes with no SNP in the probe (filled circles) and probes with a SNP in the probe (open circles)

The *maskBAD* algorithm recently published by Dannemann and colleagues [10] attempts to identify probes with SNP hybridization problems through a statistical model applied to replicates of each founder strain. To compare *equalizer* to *maskBAD*, I trained *maskBAD* on previously published microarray measurements of mouse skin from four inbred FVB/N and four inbred SPRET/Ei mice. *maskBAD* was highly sensitive, assigning low quality scores to most probes bearing a SNP, and high quality scores to most probes lacking a SNP (Fig. 3a and b). As expected, probes assigned high probe quality scores by *maskBAD* despite the presence of a SNP verified by sequence analysis were more likely to be those reporting low expression levels (Spearman rho = −0.48, *P* < 1 x 10^−16^). These probes would usually be eliminated from downstream analysis due to low signal quality. However, a significant minority of probes expressed above background levels and bearing SNPs would not be excluded by *maskBAD*. Figure 3c plots the number of above-background probes bearing or lacking known SNPs compared to their *maskBAD* quality score. Of the 378,858 probes in probesets that were expressed above background levels, 298,724 (79 %) do not intersect a SNP. 269,113 probes (71 %) had a *maskBAD* quality score above 0.2. At this stringency level *maskBAD* would remove 70,312 (19 %) probes that lack a known SNP and retain 40,701 (11 %) probes that that bear a known SNP.

### Discussion

*Equalizer* significantly reduced the effect of SNP bias, although the correction was not complete. It is plausible that the remaining deviation from the expected value of 50 % is due in part to the fact that this analysis corrected only for the presence of SNPs and did not account for other variants such as insertions, deletions, or germline copy number variations which could also affect gene expression levels. There are also large gene families such as the hair follicle keratins which may be expressed systemically at higher or lower levels in one species, and this would affect the predicted effect of a single FVB/N allele for those genes.

*MaskBAD* showed strong specificity and sensitivity if the correct quality score cut-off was chosen, and for situations where the required data are available to train its model, *maskBAD* is a valuable tool. Choosing a stringent *maskBAD* quality cut-off would result in high sensitivity and specificity, but the best value for this parameter is hard to assess without the parental genome sequences. The effect of SNPs on probe hybridization is complex, and some SNPs which intersect a probe will have no detectable effect on probe hybridization. This is more likely to be true when SNPs are located near the end of a probe rather than near the middle. Particularly in the case of genes with multiple isoforms, the effect of a given probe on the reported gene expression levels can vary between different tissues. A conservative approach which removes potentially compromised probes even if they are not obviously a problem in a particular tissue is therefore a reasonable choice. A future development step for *equalizer* will be to generate a version of the software entirely as an R package to avoid the requirement for calling a command-line script.

## Conclusions

The *equalizer* package makes it straightforward to remove individual probes from Affymetrix annotations by reading directly from VCF files describing genomic features such as SNPs which may affect cDNA hybridization. This reduces prevalence of false-positive findings in eQTL mapping studies. Importantly, this correction also increased the statistical significance of some eQTL, indicating that equalizer both reduced false positive signals and allowed results previously obscured by SNP bias to be detected. *equalizer* provides a complementary approach to statistical methods such as *maskBAD*, and it can be applied in situations such as human experiments where complete parental gene expression measurements are unavailable.

## Methods

### Sequence and microarray acquisition and normalization

A VCF file for FVB/N and SPRET/Ei mouse genomes using the MM10 annotation was downloaded from the Sanger Mouse Genomes project, *http://www.sanger.ac.uk/resources/mouse/genomes*. The backcross design ensured that all SNPs were present at or near a 50 % minor allele frequency. VCF files listing polymorphic loci in Caucasian- and African-derived populations using the HG19 annotation generated by the 1000 Genomes Project were downloaded from the NCBI Trace Archive May 2 2013 release, *ftp-trace.ncbi.nih.gov* [18]. Human variants were included if their minor allele frequency was reported as ≥ 5 % within their ethnic group. Raw microarray and human genotype data were obtained from GEO (Human: GSE24277; Mouse: GSE46077, GSE12248). Mouse genotypes were downloaded from *http://davidquigley.com/reproduce.html*. Microarray data were normalized using the *oligo* package in *R* and the Platform Design Information packages available from *bioconductor.org* [12, 21]. These packages were customized by *equalizer* as described in the text. Microarray batch effects were removed using ComBat [22]. Affymetrix platform description files using annotations that matched the VCF files were obtained from *http://www.affymetrix.com*.

### Statistical analysis

Statistical analysis was performed with *R* [21] and the *eqtl* program, a software package written in C++ that performs linear regression of gene expression compared to genotype. In analysis of human samples, *cis*-eQTL were calculated using a window of one megabase around the transcription start site for each gene. The *eqtl* package can perform *cis*-only or genome-wide eQTL analysis and can run as a multi-threaded program to exploit multiple-core computational clusters. Binary packages for *eqtl* are available at *davidquigley.com*, and *eqtl* and *equalizer* source code is freely available at *https://github.com/DavidQuigley/QuantitativeGenetics*. Source code to reproduce the analysis presented in this manuscript is available at *http://davidquigley.com/reproduce.html**.*

## Availability and requirements

**Project name:***equalizer*

**Project home page:***http://github.com/DavidQuigley/QuantitativeGenetics/tree/master/equalizer*

**Operating system:** platform independent

**Programming language:** Python, R

**Other requirements:** Bedtools 2.15 or higher

**License:** Apache 2.0

**Any restrictions to use by non-academics:** no restrictions

## Abbreviations

BED: Browser Extensible Data
SNP: Single Nucleotide Polymorphism
eQTL: expression Quantitative Trait Locus
cDNA: complementary Deoxyribonucleic Acid
VCF: Variant Call Format
mRNA: messenger Ribonucleic Acid
